# Automated system to acquire fluorescence, polarization and anisotropy maps within liquid flows

**DOI:** 10.1155/S146392460200007X

**Published:** 2002

**Authors:** Cristina M. Quintella, Cristiane C. Gonçalves, Iuri Pepe, Angelo M. V. Lima, Ana Paula S. Musse

**Affiliations:** 1 Instituto de Química Universidade Federal da Bahia Campus de Ondina Salvador-BA 40.170-290 Brazil; 2 Instituto de Física Universidade Federal da Bahia Campus de Ondina Salvador-BA 40.210-340 Brazil; 3 LPCC-College de France Paris France

## Abstract

Maps of polarization and anisotropy can be helpful for flow analysis systems (FIA, CFA, etc.) with reactions dependent on the intermolecular alignment as well as for dispersion control. Maps can be acquired manually, but when a scan over a sample area is required, the acquisition becomes tiresome and has low precision. The paper describes an automatic flexible system for high-precision sample positioning with closed loop self control, remote data acquisition and storage controlled by a BASIC program. The system was developed to acquire maps up to 850 mm^2^ of the sample (liquid flows, solids, interfaces, etc.), with up to 100 μm^2^ precision. To evaluate the equipment, performance is presented as the scan of a thin liquid film of monoethylene glycol (MEG) flowing on borosilicate. Tests were performed with and without surfactantes at submicellar concentrations: two concentrations of sodium dodecyl sulphate (SDS) and one of polyethylene oxide (PEO). For pure MEG, the intermolecular alignment initially increased, then decreased. When SDS was added, both polarization and anisotropy only increased progressively with the flow. This might be explained by the surfactant decrease of interfacial interaction. When PEO was added, both polarization and anisotropy decreased pronouncedly over the entire map, which might be due to macromolecular aggregates within the bulk generating misaligned molecular domains. The system presented as sample positioning repeatability of 0.1% and a high polarization reproducibility (error margin < 6 in 1000).


Journal of Automated Methods & Management in Chemistry
Vol. 24, No. 2 (March–April 2002) pp. 31–39

### Automated system to acquire fluorescence, polarization and anisotropy maps within liquid flows

**Cristina M. Quintella[1]*, Cristiane C. Gonçalves[1], Iuri Pepe[2,3], Ângelo M. V. Lima[1] and Ana Paula S. Musse[1]**

[1]*Instituto de Química, Universidade Federal da Bahia, Campus de Ondina, 40.170-290, Salvador-BA, Brazil* [2]*Instituto de Física, Universidade Federal da Bahia, Campus de Ondina, 40.210-340, Salvador-BA, Brazil* [3]*LPCC-College de France, Paris, France*

*Maps of polarization and anisotropy can be helpful for flow analysis systems (FIA, CFA, etc.) with reactions dependent on the intermolecular alignment as well as for dispersion control. Maps can be acquired manually, but when a scan over a sample area is required, the acquisition becomes tiresome and has low precision. The paper describes an automatic flexible system for high-precision sample positioning with closed loop self control, remote data acquisition and storage controlled by a BASIC program. The system was developed to acquire maps up to 850 mm$^2$ of the sample (liquid flows, solids, interfaces, etc.), with up to 100 $\mu m^2$ precision. To evaluate the equipment, performance is presented as the scan of a thin liquid film of monoethylene glycol (MEG) flowing on borosilicate. Tests were performed with and without surfactantes at submicellar concentrations: two concentrations of sodium dodecyl sulphate (SDS) and one of polyethylene oxide (PEO). For pure MEG, the intermolecular alignment initially increased, then decreased. When SDS was added, both polarization and anisotropy only increased progressively with the flow. This might be explained by the surfactant decrease of interfacial interaction. When PEO was added, both polarization and anisotropy decreased pronouncedly over the entire map, which might be due to macromolecular aggregates within the bulk generating misaligned molecular domains. The system presented as sample positioning repeatability of 0.1% and a high polarization reproducibility (error margin $<$ 6 in 1000).*

### Introduction

Usually, fluorescence spectroscopy monitors the sample in a single spot as a function of several factors like wavelength, time, incident light intensity, temperature, etc. This is the case of typical analysis methods like flow injection (FI) [1] and enzyme-linked immunosorbent assays (ELISA) [2]. For some applications, it is also necessary to map the sample by acquiring the fluorescence at several points.

Most of the work in the literature studies laser-induced fluorescence (LIF). Nevertheless, some applications require the knowledge of the intermolecular alignment within the sample, as in the case of tensions shear stress during resin extrusion within polymer melts [3] and of dynamic interfacial tension dependence of a liquid flowing on surfaces with different chemical constitution [4]. This information can be obtained by fluorescence depolarization.

Although there are several works in the literature that use fluorescence to monitor liquid flows as the flow progresses, those that study intermolecular alignment are not many. Kenyon *et al.* [5, 6] and Quintella *et al.* [7, 8] reported for the first time steady-state fluorescence depolarization maps of thin liquid flows. They used a non-automated sample positioning with vertical and horizontal resolution of 0.25 and 0.05 mm, respectively. The operator visually acquired their data. Owing to the high amount of points in each map, the manual acquisition was lengthily and tiresome, and could take up to 12 h.

The present paper presents a fully automated laser spectrometer, which was developed to acquire high-resolution maps of intermolecular alignment (polarization and anisotropy) and total fluorescence, over a predetermined area of liquid flows, solids and interfaces.

Here is presented, as an application of the system developed, the flow on borosilicate of a thin liquid sheet of monoethylene glycol (MEG) with and without submicellar concentrations of surfactants.

Surfactants are present in several flow systems, such as in waste waters [9] where they may become environmental contaminants. They can be used to improve chemical analysis, sampling and storage like in polycyclic aromatic hydrocarbons (PAHs) in water [10] and may interfere in sample recovery. Their hydrophobic–hydrophilic properties make them perfect solubilizers for mixtures of polar and non-polar compounds, avoiding precipitation. Analytical methods may use surfactants with concentration either below or over critical micellar concentration (CMC), like micellar extraction [11], and enhancement of neutral analytes separation [12], which determination may involve flow injection analysis (FIA) [9, 13]. In fluorescence spectroscopy, surfactants increase the quantum yields of fluorimetric sensors [14, 15] as well as enhancing chemiluminescence of low fluorescent reagents [16]. There are some rheological studies at premicellar and micellar concentrations [17] that have studied the flow evolution at a single point.

Although surfactants are present in a wide range of chemical analysis processes, their contribution to the intermolecular orientation within the flow have only recently been addressed more thoroughly.

*To whom correspondence should be addressed. e-mail: cristina@ufba.br

### Theory

The theory has been presented before [5, 6]. Briefly, vertically polarized laser light excites the fluorescent probes within the sample. Their absorption is proportional to the cosine square of the angle between the molecular dipole and the laser electric field. If the dipole is parallel to the longitudinal molecular axis, mainly nearly vertical molecules will absorb laser radiation and a photoselection of the probe occurs.

Fluorescence detection can be done by two laser spectroscopic techniques: Laser induced fluorescence (LIF) and fluorescence depolarization or polarized laser-induced fluorescence (PLF) [4, 6]. In LIF the fluorescence emission is collected by a detection system regardless to its polarization and the data give direct information on how the probes align upon excitation. When the sample is a liquid flowing vertically with shear imposed intermolecular alignment, the LIF signal is proportional to the fraction of molecules aligned along the flow direction at the excitation moment.

In PLF, the fluorescence emission is discriminated according to its polarization and compared with the laser polarization [5, 6]. While excited, the photoselected probes can rotate or not as a function of the chemical environment. When fluorescence takes place, the light emitted is not 100% polarized as the probe transition dipole moment will depend on the molecular orientation of the probe.

The intermolecular alignment data can be interpreted either as a bidimensional phenomenon in terms of polarization (P) [5, 6], or as a three-dimensional phenomenon in terms of anisotropy ($r$) [18]:

$$P = \frac{I_{\parallel} - I_{\perp}}{I_{\parallel} + I_{\perp}} \qquad r = \frac{I_{\parallel} - I_{\perp}}{I_{\parallel} + 2I_{\perp}}.$$

Anisotropy $r$ will depend on the local rotation diffusion time $\phi$ [19]. For steady-state fluorescent depolarization it is given by:

$$r_0/r = 1 + \tau/\phi, \qquad \phi = \eta V/RT,$$

where $r_0$ is the initial anisotropy, $\tau$ is the lifetime of the excited state, $\eta$ is the viscosity, $V$ is the volume rotating unit, $R$ is the gas constant and $T$ is the temperature.

If the excited state lifetime is of the same order of the probe rotational diffusion, then fluorescent probes become efficient intermolecular alignment sensors. Thus, the fluorescent species behaves as a medium of varying birefringence that permits molecular-level observation of fluid flow, the birefringence being particularly sensitive to the velocity gradient within the fluid.

### Experimental

The equipment is an ensemble of mechanical hardware, electronic hardware, optic devices, a light detection system and software. The main idea here was to develop a technological tool capable of a high level of flexibility and precision in sample positioning, closed loop self control, and high-density data acquisition and storage.

### *Automatic sample positioning system*

The spectrometer can scan samples up to 30.9 mm wide and 27.4 mm long, covering a $\sim 850\,\text{mm}^2$ surface, with a fine precise path across the stream ($X$) and down stream ($Y$). This constraint imposes the need of an especially high precision and high repeatability mechanical hardware, allied to a flexible positioning control software.

At each point, the intermolecular alignment data is automatically acquired.

The mechanical design uses two perpendicular stainless-steel endless-screws assembled in a brass frame. The screws are driven by two independent (1.8°) step-motors (1 and 2 in figure 1A) with 10 μm precision in each direction. The set-up positioning repeatability was determined by successive back and forth cycles. At each cycle end, the distance to the mechanical frame origin was determined using a digital calliper, being the positioning repeatability $> 0.1\%$.

The sample holder was designed to be a very open and flexible structure. The simplicity in its mechanical design allows for the improvisation of different holding options, allowing the sample adjustment over 3 degrees of freedom: $Y$, angle in the $XZ$ plane and the angle in the $YZ$ plane.

The electric signals driving and controlling the step-motors were generated by the data acquisition and control system (see below). An interlock system prevents any potential damaging caused by an improper manoeuvre.

### *Electronic hardware*

The electronic hardware can be divided in two parts. The first comprehends the two step-motors' interface. Figure 2 shows the command latch, the power drive outputs and the interlock buffer input. A software real-time driver was chosen to drive the motors instead of an onboard hardware driver. This choice was based on the search for a high flexibility interfacing system, allowing different drive options (full step, half step, low and high torque). This has been possible as the output from the photodiode (the fluorescence detector) is a slow signal, leaving a long no operation delay between consecutive measures.

The second part of the electronic hardware consists on an analogue section where a high impedance low noise amplifier prepares the fluorescence signals to be digitized. The digitalization was performed by a Burr Brown ADS7804, 10 μs, analogue-to-digital converter (ADC) [20]. A logic chip set allowed communication with a personal computer (PC) by the standard parallel printer port [21].

### *Light detection system*

Figure 3 shows a scheme for the PLF experimental set-up.

A Coherent Inova60 argon laser generated the 514.5 nm light beam. The laser intensity was kept at 50 mW, a safe

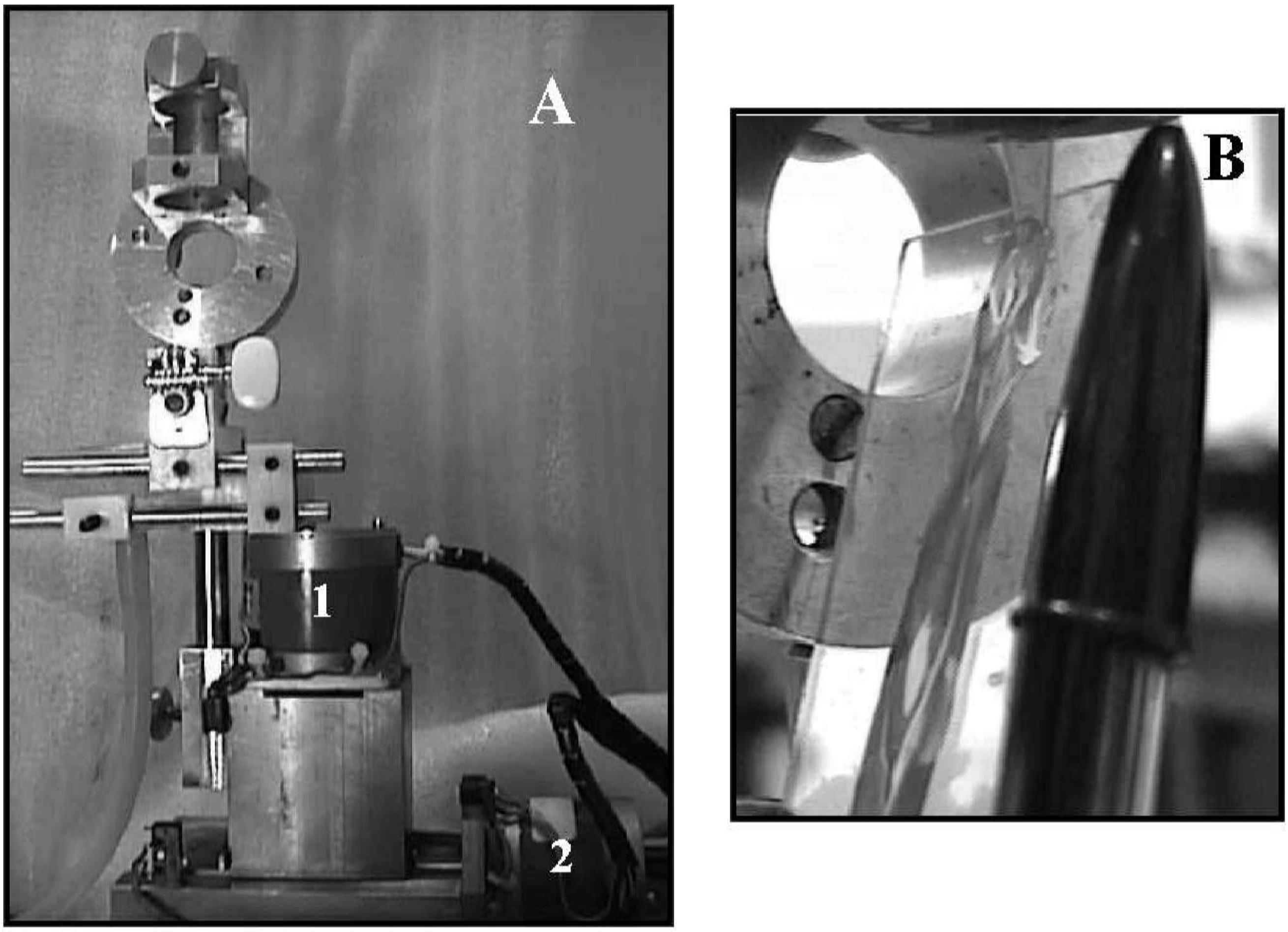

*Figure 1. (A) Mechanical hardware; (B) sample holder showing a thin sheet of liquid MEG flowing on borosilicate.*

value as saturation phenomena started over 80 mW. The laser light was deflected by a mirror (E) and focused into the sample (SPS), on a 0.02 mm$^2$ diameter spot, by a biconvex borosilicate lens ($L_1$) with a 400 mm focal length. A vertical Glan-Thompson polarizer ($P_1$) guarantees 100% polarization. The fluorescent probes within the sample absorb the laser radiation. After a given time delay, depending on their excited state lifetime, they fluoresce. The fluorescence is collected by a biconvex borosilicate lens ($L_2$), with 50.8 mm focal length, being focused into the photodiode (PD) at a 0.02 sr solid angle. A 550 nm high-pass filter (F) blocked the non-absorbed laser light, avoiding light mixing and PD overflow.

To measure both vertical ($I_{\|}$) and horizontal ($I_{\perp}$) fluorescence components with the same optical set-up, the Conoptics 350-50 photo-elastic modulator (PEM), driven by a sinusoidal generator (G) at 600 Hz, periodically rotated the fluorescence light electric axis around the propagation axis ($Z$). The PEM-modulated output light beam was selected by the horizontal Glan-Thompson polarizer ($P_2$). The PD detected an alternated signal $V_{AC}$ superposed to a continuous signal $V_{DC}$.

The OPT301 Burr Brown photodiode [22] is an opto-electronic integrated circuit, containing a photodiode and a transimpedance amplifier on a single di-electrically isolated chip. The integrated combination of photodiode and amplifier eliminated leakage current errors, noise pick-up and gain peaking. The light detection sensibility can be adjusted by a set of dipswitch and external resistors. The spectral responsivity is a broad curve covering from the near IR to the near UV, ensuring that the system can work in a very large light range, allowing the use of several fluorescence probes.

The PD signal was split up in two by a passive R-RC network (SB). The direct coupled portion of this signal ($V_{DC}$) goes both to a digital voltmeter ($M$) and then to ADC 2 (figure 2) in the interface ($I$). The capacitor-coupled portion of the photodiode signal $V_{AC}$ goes to the lock-in ($AS$) to be detected in phase with the reference sinusoidal PEM modulating signal. The filtered $V_{AC}$ is phase locked, amplified and then sent to ADC 1 (figure 2), in the interface ($I$), to be digitized.

$V_{AC}$ corresponds to the difference between vertical ($I_{\|}$) and horizontal ($I_{\perp}$) fluorescence components and $V_{DC}$ to their sum [5, 6]. The polarization $P$ is obtained as:

$$P = \frac{V_{AC}}{V_{DC}}\frac{1}{F_C} \qquad r = \frac{2P}{3 - P},$$

where $f_C$ is the correction factor due to birefringence of optical components, being 0.20 for both vertical and horizontal polarized light.

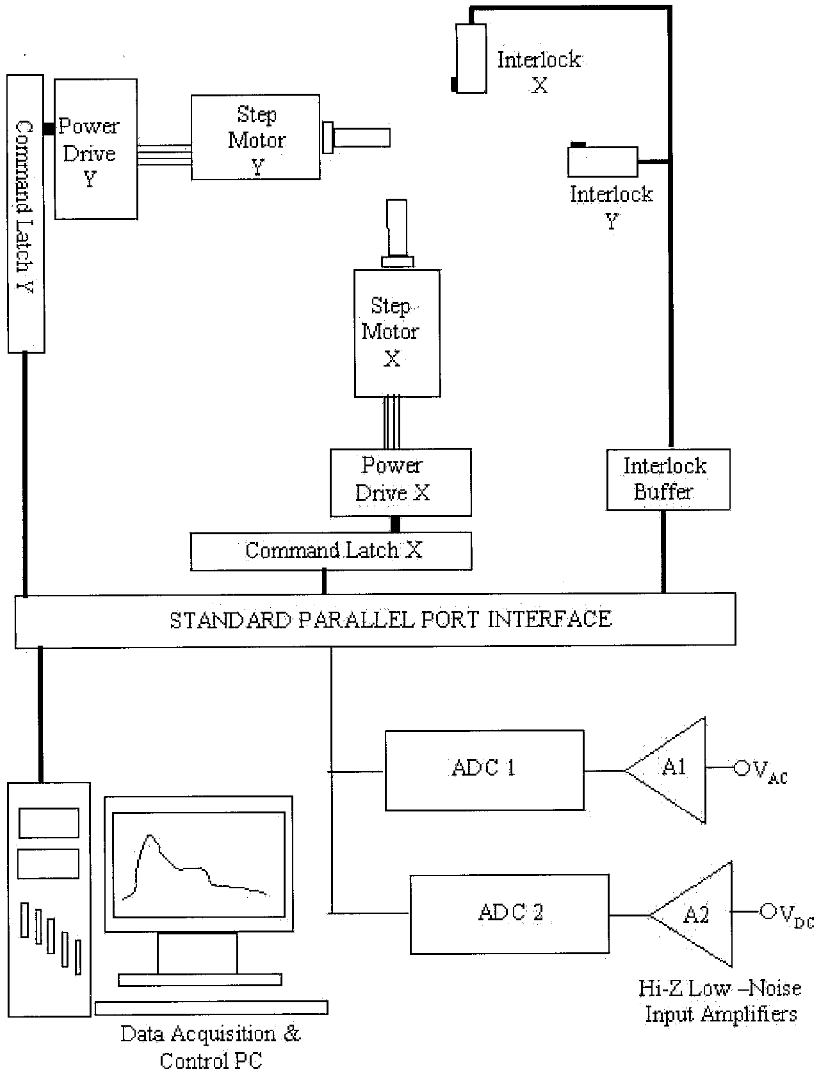

*Figure 2. Electric hardware blocks diagram showing the command latch, power drive outputs and interlock buffer input for* X- *and* Y-*step motors, as well as the analogue section for fluorescence signals digitization.*

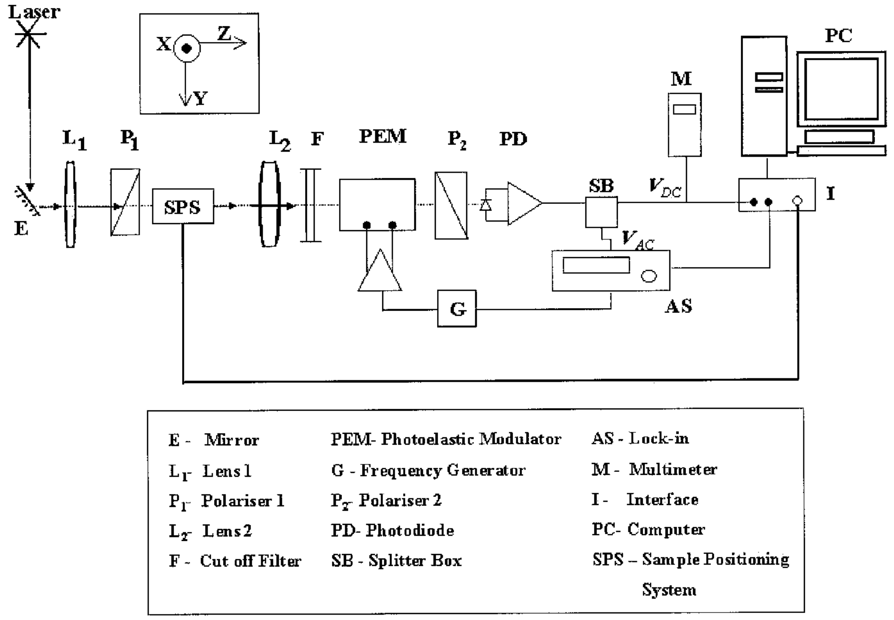

*Figure 3. Experimental set-up for polarized induced fluorescence (PLF) detection.*

Digital images can be acquired with a Hitachi VM-E230A video camera (36× zoom) connected to the PC.

*Software*

As mentioned, the concepts behind this equipment design are full automation, flexibility, precision, repeatability, easy data acquisition and reliable data storage. The PLF spectrometer was projected to be compatible with a small general propose PC and to have high level of portability.

In a hardware sense, the portability was reached using a fully IBM-PC interfacing design, specially in terms of the data communication channel, the standard Centronics parallel port (SPP) [23].

In a software sense, the conception scenario is a little different. Although Microsoft Windows has been the leader for about 15 years, Linux now menaces its position. We have chosen an interpreted programming language, trying to be independent of a specific compiler. The code can be interpreted by the BASIC for MSDOS or, eventually, by BASIC for Linux.

Figure 4 shows the simplified software block diagram as the program itself has more than 500 code lines. In the start-up routine, the program searches for the SPP location in the PC IO-map and starts the communication protocol. All constants and positioning parameters are loaded up, passing to the auto-zeroing phase for both motors. Then, the program exhibits a dialogue screen asking for one of the operation choices: spectrum acquisition or motor displacement to an arbitrary position or a fixed point, long-term data acquisition.

The spectrum acquisition routine starts showing a graphic screen (intensity versus position). During data acquisition, there are different options in the operation mode: measuring delay, channel 1 gain, channel 2 gain, numerical off-set, number of steps, $X$ increment and $Y$ increment. This interactive screen is used as a monitoring tool, where one can visualize in a rough way the data being acquired, i.e. $V_{AC}$ and $V_{DC}$, and decide to save, or not, a given dataset. The acquisition routine is not a final data-analysing facility as the saved data should be the object of post-data treatment and analysis.

The motor displacement routine starts loading up the present position and asks for the new location. In that sense, it is almost a manual operation mode, where one can bias the $XY$ frame origin. This routine is particularly useful during the experiment preparation and adds many flexibility and test possibilities. Throughout the duration of any motor displacement, the operation can be stopped and the automatic zeroing can be started.

The third option is the fixed-point long-term data acquisition as a function of time. The subroutine starts asking for a position and, once there, keeps taking data. The operator determines the position and acquisition time intervals.

*Data reproducibility and control tests*

Polarization and anisotropy were monitored as a function of liquid flow temperature, PEM modulation frequency and acquisition time at a fixed position.

A temperature increase produces an initial signal increase, stabilizes at a maximum value and then decreases (figure 5A, C). When the temperature is changed, the dye probe rotational diffusion is also varied. For fluorescence depolarization, the ideal value is when the rotational diffusion of the probe is of the same order of its excitation lifetime (see above). This is achieved between 14 and 17 °C.

The polarization fluctuation with time was obtained during a control test, at a fixed position, and repeated for several polarization intensities. The test at 5% polarization (0.03 anisotropy) took 500 s and gave $(1069 \pm 6) \times 10^{-4}$ (figure 5B, D). The test at 15% polarization took 1180 s and gave $(15.22 \pm 0.09)\%$.

The anisotropy and polarization response to modulation frequency was monitored from 10 to 1000 Hz. The frequency 600 Hz was chosen to avoid noise from other electrical systems.

The manual scan had a resolution of (0.35 mm), whilst the automatic had 100 $\mu m^2$. The sample positioning repeatability was better than 0.1%.

To validate the system, the first lobe of a liquid thin jet was scanned in the same conditions as those presented by Quintella *et al.* [8].

### Application

The sample system (figure 1B) consisted of a liquid thin sheet flowing on a Perfecta borosilicate microscope slide ($75 \times 25 \times 0.8$ mm, roughness $< 5$ nm) at 80° with the horizontal. Before the experiment, the slide was kept for 10 min in an ultrasound bath with ethanol and dried for 15 min in a stove at 50 °C.

Ethylene glycol (MEG) (Merck 10921, 99.5% purity) passed through a polished sapphire slit nozzle with 100 μm ×6 mm, generating a thin free jet and impinging perpendicularly on the solid surface. The liquid flowed at 220 cm $s^{-1}$ constant average velocity. Its thickness ranged from 150 μm at the middle to 1000 μm at the borders. A home-made thermal bath kept the temperature at $15.0 \pm 0.5$ °C.

Rhodamine 6G (R6G) (Lambdaphysik, 99.99% purity) was used as fluorescent probe. It has transition dipole moment parallel to the molecule longitudinal direction [24] and was pumped from state $S_0$ to $S_1$ [25]. The concentration of $1.9 \times 10^{-3}$ mol $l^{-1}$ was chosen to maximize the signal and avoid quenching processes due to excitation migration like Föster transfer [26]. Under this experimental condition, the viscosity was 0.225 g $cm^{-1}$ $s^{-1}$. The rotational diffusion time and excitation lifetime are in the order of 4.2 and 4.9 ns, respectively [27].

The influence of two classes of surfactants as well as concentration was studied. The detergent sodium dodecyl sulphate (SDS) (Sigma L-6026, > 99% purity) was added to MEG at two concentrations ($0.35 \times 10^{-3}$ and $4 \times 10^{-3}$ mol $l^{-1}$). The long chain (high molecular weight) ethylene polyoxide (PEO) ~4 000 000 (Aldrich 18,946–4) was added at $3.5 \times 10^{-3}$ mol $l^{-1}$. All surfactant

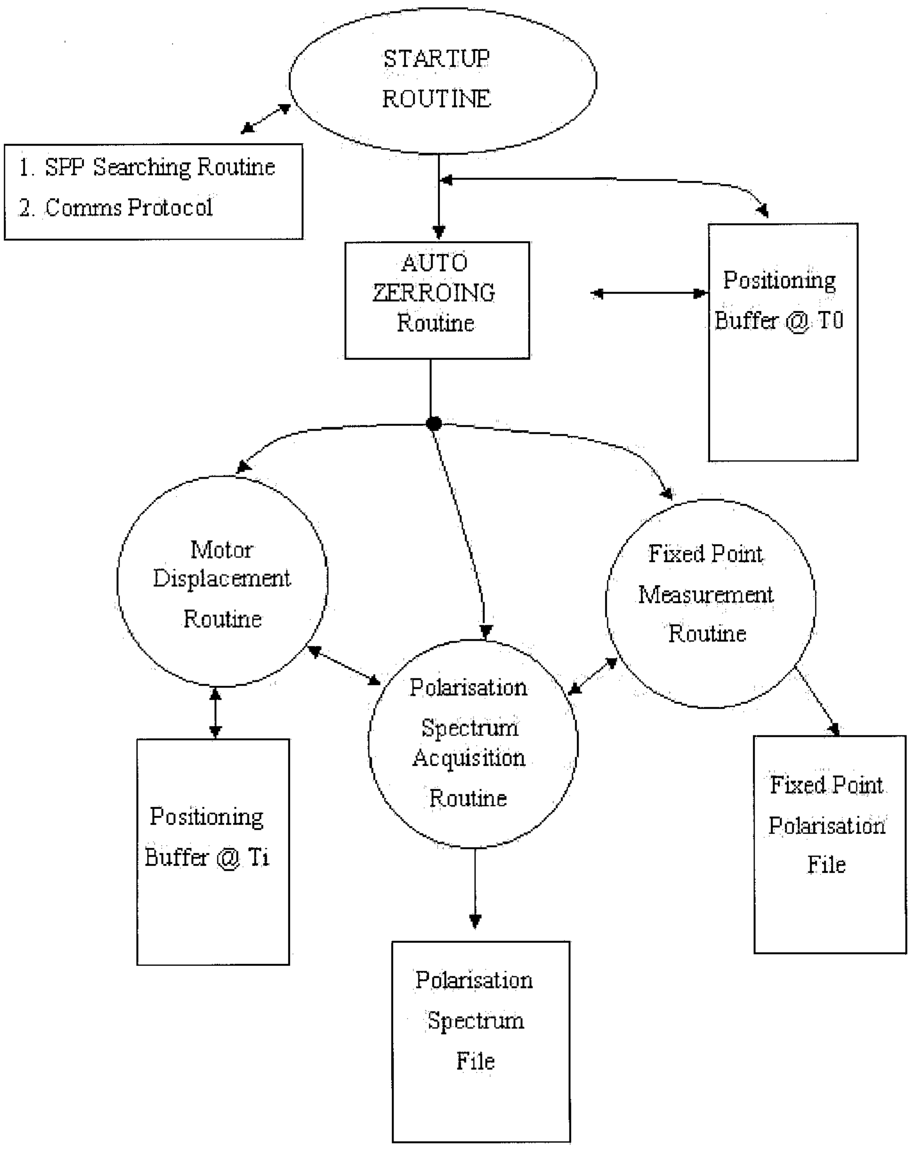

*Figure 4. Simplified software block diagram for sample positioning and remote fluorescence signals acquisition ($V_{AC}$ and $V_{DC}$).*

concentrations were submicellar as determined by conductivity and surface tension measurements.

Figure 6 presents the maps acquired. Each polarization and anisotropy map was acquired over 134.4 $mm^2$ with 1.40 $mm^2$ resolution (a total of 119 data points).

Both SDS and PEO increased the liquid sheet width. It is well known [28] that surfactants decrease the liquid–solid interfacial tension, becoming good wetting agents. This effect is more pronounced in dynamic systems, as opposite to static, as surfactants tend to migrate to the surfaces, increasing their concentration locally.

Within pure MEG (figure 6A), there is an initial decrease in polarization and anisotropy, followed by an increase as the flow progresses.

The initially low MEG polarization and anisotropy may be due to strong interfacial interactions with borosilicate [4]. The liquid has hydrogen atoms and hydroxyls, whilst the solid surface is composed by highly electronegative atoms and has been cationized. It is likely that hydrogen bond-type interactions are established between the liquid and surface, slowing the molecules flowing near the interface and causing local microturbulences, i.e. misaligned molecular domains [29]. As MEG has strong intermolecular bonds, these turbulent domains may have a medium range within the liquid, decreasing the intermolecular alignment at the bulk. Further down the stream, this effect is overcome and the intermolecular alignment increases.

When SDS is added (figure 6B), both polarization and anisotropy increase monotonically as the liquid flows. The interfacial tension is lowered by the surfactant. Thus, the molecules flowing near the interface will have weaker interfacial interactions, becoming more aligned with the flow. It is even possible that a certain degree of slip velocity occurs at the interface. It is likely that the dispersion [30] will also decrease as the molecular domains become more laminar. When the SDS concentration is increased (figure 6C), the polarization and anisotropy stay almost identical. This may be due to

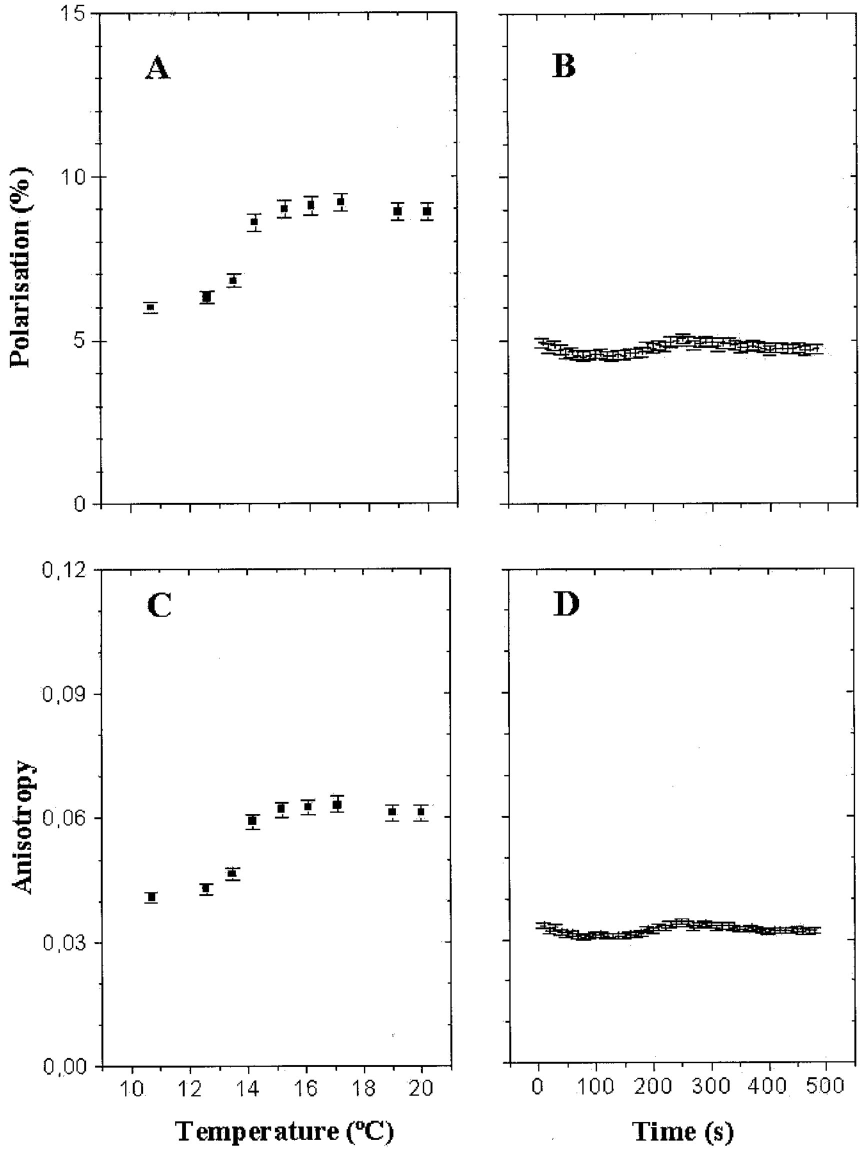

*Figure 5. Control tests for polarization (A, B) and anisotropy (C, D) reproducibility as a function of liquid flow temperature (A, C) and time fluctuation at the same sample spot (B, D).*

the interacting sites of the interface being already saturated by surfactant molecules, thus this concentration increase did not cause significant effects.

When the solute is PEO (figure 6D), the interfacial tension is also lowered, which should increase polarization and anisotropy. Similarly to SDS, the long-chain PEO may interact with the borosilicate at several points of the same molecule, generating misaligned molecular domains at the interface and causing the molecules flowing near the interface to be more turbulent. Nevertheless, a wide and nearly homogenous region of low values replaces the polarization and anisotropy gradation seen with SDS. This may be explained by the PEO long chains within the bulk being in between the small MEG molecules, disturbing the intermolecular bonds network and misaligning the molecular domains. It is also well known that long-chain polymers like PEO tend to form molecular aggregates [28]. These structures may also decrease significantly the intermolecular alignment of the MEG flow.

### Conclusion

The automatic system described here for sample *XY* positioning and remote acquisition of polarization and anisotropy made it possible to acquire higher definition maps to reduce acquisition time and increase data reproducibility and resolution.

The intermolecular alignment of a thin sheet of liquid MEG flowing on borosilicate, evaluated through polarization and anisotropy maps, decreased initially due to strong interfacial tension, thus increasing the misaligned molecular domains and consequent dispersion.

Addition of the surfactant SDS at two submicellar concentrations increased the intermolecular alignment progressively with the flow. This may be explained by the decrease interfacial tension, allowing the molecular domains to align themselves with the flow.

Addition of the long-chain polymer PEO decreased pronouncedly the intermolecular alignment. This may

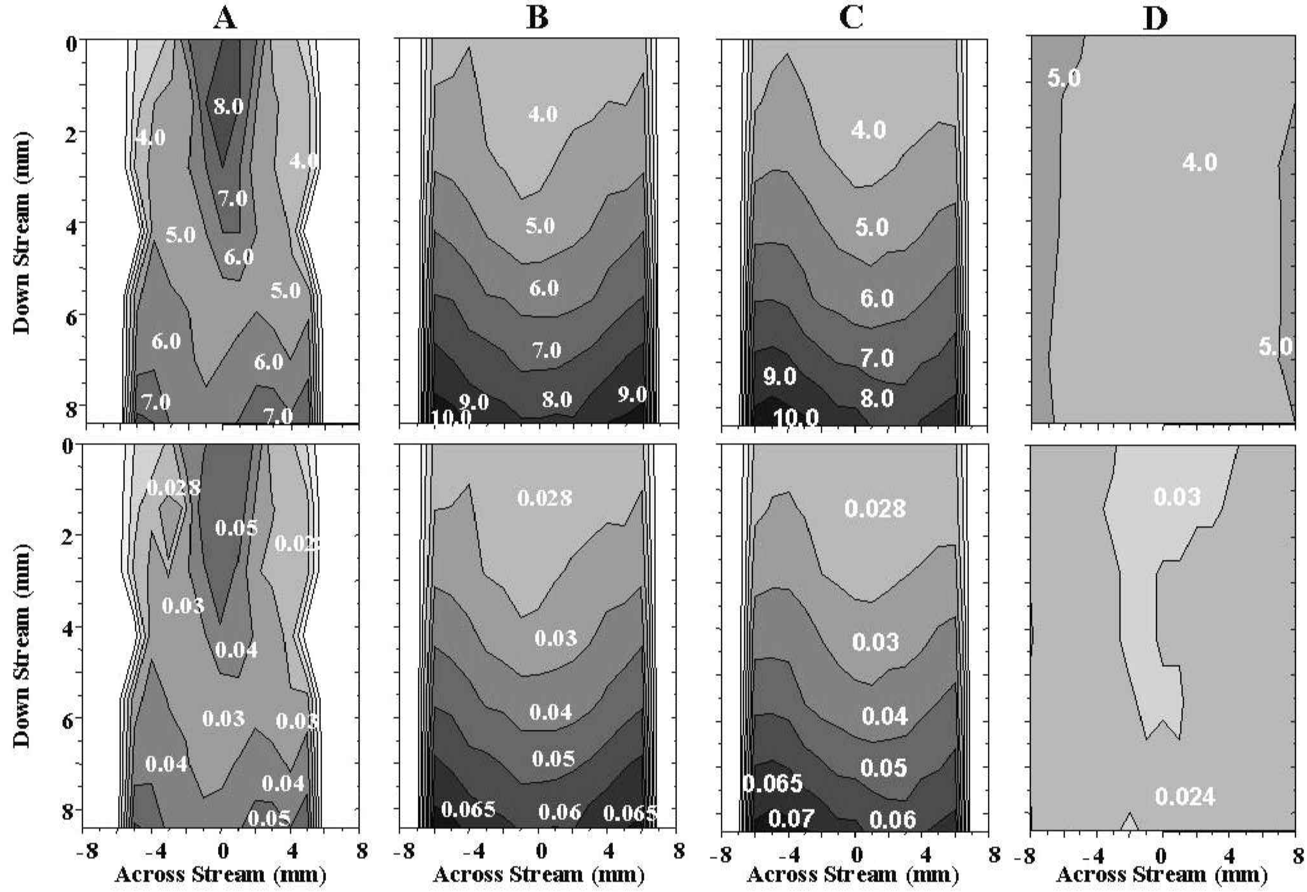

*Figure 6. Intermolecular alignment maps of polarization (top) and anisotropy (bottom) obtained for: (A) pure liquid MEG flow; (B)* $0.35 \times 10^{-3}$ *mol l*$^{-1}$ *SDS surfactant solution; (C)* $4 \times 10^{-3}$ *mol l*$^{-1}$ *SDS surfactant solution; and (D)* $3.5 \times 10^{-3}$ *mol l*$^{-1}$ *PEO surfactant solution.*

be due to formation of polymer-aggregated structures within the bulk-generating misaligned molecular domains.

Flow analysis systems, with intermolecular alignment-dependent reactions, that make use of surfactants may have their values altered by the non-isotropic intermolecular alignment distribution of the molecular domains, and their subsequent dispersion efficiency.

### Acknowledgements

Grants from the Conselho Nacional de Desenvolvimento Científico e Tecnológico (CNPq, Brazil) and PADCT3 supported the work. A. J. McCaffery is thanked for providing the sapphire slit nozzle. A. M. V. L. and A. P. S. M. acknowledge their undergraduate research fellowship from PIBIC-CNPq and CNPq. C. C. G. acknowledges a DPhil scholarship. C. M. Q. acknowledges a senior research scholarship from CNPq.

### References

1. Wells, I. and Worsfold, P. J., *Journal of Automated Methods and Management in Chemistry*, **72** (1999), 113.
2. Rhee, J. I., Hagedorn, J., Scheper, T. and Schugerl, K., *Journal of Automated Methods and Management in Chemistry*, **21** (1999), 121.
3. Bur, A. J., Roth, S. C. and Thomas, C. L., *Review of Scientific Instruments*, **71** (2000), 1516.
4. Quintella, C. M., Gonçalves, C. C., Pepe, I., Lima, A. M. V. and Musse, A. P. S., *Journal of the Brazilian Chemical Society*, **12** (2001), 780.
5. Kenyon, A. J., McCaffery, A. J. and Quintella, C. M., *Molecular Physics*, **72** (1991), 965.
6. Kenyon, A. J., McCaffery, A. J., Quintella, C. M. and Winkel, J. F., *Molecular Physics*, **74** (1991), 871.
7. Quintella, C. M., Gonçalves, C. C., Musse, A. P. S. and McCaffery, A. J., *Experiments in Fluids* (submitted).
8. Quintella, C. M., Gonçalves, C. C., Musse, A. P. S. and McCaffery, A. J. (in preparation).
9. Bruzzoniti, M. C., Sarzanini, C. and Mentasti, E., *Journal of Chromatography A*, **902** (2000), 289.
10. Brouwer, E. R., Hermans, A. N. J., Lingeman, H. and Brinkman, U. A. Th., *Journal of Chromatography A*, **669** (1994), 45.
11. Maniasso, N., *Química Nova*, **24** (2001), 87.
12. Wang, X. and Bobbitt, D. R., *Talanta*, **53** (2000), 337.
13. Lucy, C. A. and Tsang, J. S. W., *Talanta*, **50** (2000), 1283.
14. Choi, M. M. F. and Tse, O. L., *Analytica Chimica Acta*, **423** (2000), 229.
15. Louro, S. R. W., Nascimento, R. O. and Tabak, M., *Biochimica et Biophysica Acta*, **1190** (1994), 319.
16. Zhang, G. and Chen, H., *Analytica Chimica Acta*, **409** (2000), 75.
17. Kim, W. J. and Yang, S. M., *Journal of Colloid and Interface Science*, **232** (2000), 225.
18. Kawski, A., *Critical Reviews in Analytical Chem*istry, **23** (1993), 459.
19. Lakowicz, J. R., *Principles of Fluorescence Spectroscopy* (New York: Plenum, 1983).
20. *IC Data Book: Data Conversion Products* (Tucson: Burr-Brown, 1995).
21. Grade, D. V., *Programming the Parallel Port* (Saint Lawrence: R & D Books/Miller Freeman, 1998).
22. *IC Data Book: Mixing Signals Product* (Tucson: Burr-Brown, 1998).

23. Axelson, J., *Parallel Port Complete: Programming, Interfacing and Using the PC's Parallel Printer Port* (Madison: Lakeview Research, 1996).
24. Penzkofer, A. and Wiedmann, J., *Optics Communications*, **35** (1980), 81.
25. Aristov, A. V. and Shevandin, V. S., *Optics and Spectroscopy*, **44** (1978), 131.
26. Knox, R. S., *Physica*, **39** (1968), 361.
27. Scully, A. D., Matsumoto, A. and Hirayama, S., *Chemical Physics*, **157** (1991), 253.
28. Adamson, A. W. and Gast, A. P., *Physical Chemistry of Surfaces* (New York: Wiley, 1997).
29. Juzeliunas, G. J., *Journal of Luminescence*, **46** (1990), 201.
30. Narusawa, Y. and Miyamae, Y., *Talanta*, **45** (1998), 519.

